# Obesity: A Chronic Low-Grade Inflammation and Its Markers

**DOI:** 10.7759/cureus.22711

**Published:** 2022-02-28

**Authors:** Deepesh Khanna, Siya Khanna, Pragya Khanna, Payal Kahar, Bhavesh M Patel

**Affiliations:** 1 Foundational Sciences, Nova Southeastern University Dr. Kiran C. Patel College of Osteopathic Medicine, Fort Lauderdale, USA; 2 Pediatrics, Gujarat Medical Education and Research Society (GMERS) Medical College, Vadnagar, IND; 3 Department of Health Sciences, Florida Gulf Coast University, Fort Myers, USA

**Keywords:** role of hormones in obesity, obesity and inflammatory markers, prevalence of obesity, obesity: an immune disease, fatty acid, adipose tissue, browning of white adipose tissue, adipokine, inflammatory processes/inflammatory markers, nutrition and metabolism .obesity. dietary fiber

## Abstract

As the prevalence of obesity continues to rise, the world is facing a major public health concern. Obesity is a complex disease associated with an increase in several inflammatory markers, leading to chronic low-grade inflammation. Of multifactorial etiology, it is often used as a measurement of morbidity and mortality. There remains much unknown regarding the association between obesity and inflammation. This review seeks to compile scientific literature on obesity and its associated inflammatory markers in chronic disease and further discusses the role of adipose tissue, macrophages, B-cells, T-cells, fatty acids, amino acids, adipokines, and hormones in obesity. Data were obtained using PubMed and Google Scholar. Obesity, inflammation, immune cells, hormones, fatty acids, and others were search words used to acquire relevant articles. Studies suggest brown adipose tissue is negatively associated with body mass index (BMI) and body fat percentage. Researchers also found the adipose tissue of lean individuals predominantly secretes anti-inflammatory markers, while in obese individuals more pro-inflammatory markers are secreted. Many studies found that adipose tissue in obese individuals showed a shift in immune cells from anti-inflammatory M2 macrophages to pro-inflammatory M1 macrophages, which was also correlated with insulin resistance. Obese individuals generally present with higher levels of hormones such as leptin, visfatin, and resistin. With obesity on the rise globally, it is predicted that severe obesity will become most common amongst low-income adults, black individuals, and women by 2030, making the need for intervention urgent. Further investigation into the association between obesity and inflammation is required to understand the mechanism behind this disease.

## Introduction and background

Obesity is one of the most prevalent non-communicable diseases with major public health concerns [1]. Different methods of assessment can be utilized to calculate the various facets of obesity, such as total or regional obesity. In a clinical setting, obesity and the complications associated with it can be used as a measure to estimate morbidity and mortality, while body mass index (BMI) has been used to screen overweight (BMI=25-29.9) and obese (BMI>30) individuals. Waist circumference is the best anthropometric indicator of visceral fat and a better predictor of metabolic syndromes such as diabetes, hypertension, and dyslipidemia [2]. Individuals with a normal BMI and a large waist circumference are at higher risk of such diseases. However, combining BMI and waist circumference to assess disease state accounts for relatively less risk prediction as they are co-linear in nature. Hip circumference is a parameter inversely related to metabolic syndrome, and a large hip circumference has been linked to a lower risk of diabetes and coronary heart disease, due to the involvement of a large muscle mass. [2]

Obesity has a significant inflammatory component that is associated with alterations in insulin resistance, hypertension, atherosclerosis, and some cancers [3,4]. Individuals that are overweight and obese have altered serum levels of inflammatory cytokines such as tumor necrosis factor-alpha (TNF-𝛼), C-reactive protein (CRP), interleukins (IL-6, IL-18), resistin and visfatin [5-9]. Measures of body fat are more strongly correlated with such inflammatory markers, compared to BMI [6,8,10].

Dietary intervention, specifically caloric restriction, and regular exercise have been proven effective to reduce inflammation in obesity, and related metabolic dysfunctions [11,12]. However, dietary weight loss can be less effective as a long-term anti-inflammatory intervention [13-15]. Regular exercise is important for the treatment of chronic inflammation and obesity-related conditions such as metabolic syndrome since it has proven effective in reducing metabolic hormones [16].

It is an extensively documented phenomenon that obesity and its inflammatory markers pose significant effects on diabetes, hypertension, and other chronic conditions. This review provides a detailed insight into the prevalence and pathophysiology of obesity and the effects of inflammatory markers on chronic conditions.

## Review

Methods of literature review

English-language articles on the topics of obesity and inflammatory markers published between 1993 and 2022 were identified using the databases PubMed and Google Scholar. Search words were "obesity," "fatty acids," "adipose tissue," "inflammatory markers," "macrophages," "immune cells," "leptin," "resistin," and "visfatin." The review yielded a total of 93 articles that emphasized the description of obesity as low-grade chronic inflammation or immune disease, its inflammatory markers, specifically resistin and visfatin, and the prevalence and effect of fatty acids on inflammation.

Discussion

Brown and White Adipose Tissue

Brown fat cells and muscle cells contain similar markers due to their common derivation from the stem cells in the embryo. Brown fat, more commonly found in newborns and hibernating animals, functions to generate heat by burning calories. The amount of brown fat decreases significantly after infancy. Adults with higher levels of brown fat tend to be younger in age and have a slenderer body type along with normal blood sugar levels. Reserves of brown fat in the body can be increased by exercise, adequate sleep patterns since melatonin can influence the production of brown fat, and regular exposure to the cold, which can be achieved via performing exercise outdoors during the winter months or in a cold room [17]. On the other hand, white fat is the predominant form of fat in the body that originates from connective tissue, functions as an energy reserve in the body, a thermal insulator, and a cushion for internal organs. It also acts as an endocrine organ and produces multiple hormones that are associated with metabolic syndrome. White fat also has receptors for insulin, growth hormone, epinephrine, cortisol (stress hormone), etc. [17]. An excess of white fat throughout the body has been associated with an increased risk of cancer in the colon, breast, esophagus, gall bladder, and pancreas. It is also associated with sleep apnea and progressively debilitating conditions such as arthritis of the joints [17].

White adipose tissue (WAT) is not only an energy storage place in the body but also an important regulator of metabolic pathways including immunity and inflammation [18]. Adipose tissue releases multiple pro-inflammatory and anti-inflammatory factors such as adipokines leptin, adiponectin, resistin, and visfatin and cytokines such as IL-6, etc. [19,20]. Pro-inflammatory factors play an important role in insulin resistance and increased risk of cardiovascular diseases (CVD) associated with obesity [21]. In contrast, a decrease in leptin levels might predispose to increased predisposition to infection due to reduced T-cell responses in malnourished individuals [22]. Inflammatory conditions lead to changes in adipokine levels, although their pathogenic role has not been completely clarified. A study conducted by Vijgen et al. in 2011 showed the relationship between brown adipose tissue and both BMI and body fat percentage [23]. With an R-value of 0.8 and 0.75, respectively, the results indicated a strong negative association between brown adipose tissue activity (kBq) and both BMI and body fat percentage. As the BMI of the group approaches >30 indicating an obese status, the activity of the brown adipose tissue responsible for burning calories declined significantly. Similarly, as the body fat percentage of the group rose over 20% indicating obesity, brown adipose tissue activity declines significantly [24]. This further reinforces the notion that brown adipose tissue plays an important role in metabolism.

Obesity: An Immune Disease?

Obesity is a state of excess body fat associated with changes in neutrophil, monocyte, and lymphocyte count, as well as lower T-cell and B-cell-induced proliferation, leading to an overall decrease in immune function [25]. With this in mind, we must ask ourselves if the onset of obesity could be due to an altered immune system? This conclusion would be too speculative and not sufficiently founded, however, we can attribute immune dysfunction to be a major contributor to obesity-associated manifestations such as insulin resistance and inflammation. With an increase in the severity of obesity, macrophage aggregates grew larger as well, exhibiting a similar pattern as seen in other inflammatory conditions. This led to the hypothesis that macrophage aggregates could be used as a measure of the inflammatory state in obesity [18].

Prevalence of Obesity

Obesity, as defined by the World Health Organization, is a condition of abnormal or excessive fat accumulation in adipose tissue to the extent that health may be missing [26]. For the Western population, the current guidelines classify obesity as a BMI >30 kg/m^2^. In the United States, the prevalence of obesity has increased more than two-fold over the past 40 years. However, there continue to be great differences between certain population groups, along with continued changes in associated patterns [27-29]. The prevalence of obesity increased by 7.9% for men and by 8.9 % for women between the years of 1976-1980 and 1988-1994 for adults aged 20 to 74 years, and subsequently by 7.1% for men and by 8.1% for women during 1988-1994 and 1999-2000 [30]. The prevalence of obesity in 2007-2008 was approximately 32.2% amongst adult men, and approximately 35.5% amongst adult women [31], which in 2009-2010 increased to 35.5% in men, and 35.8% in women, respectively [32]. Data from 2011 to 2012 further suggest that among men and women aged 20 and over, 34.9% of men and 36.1% of women were classified as obese, while 39.96% of men and 29.74% of women were overweight [33]. In the past decade, adult women aged 20 and over across the United States have experienced a sharp increase in the prevalence of obesity, from 35.8% in 2009-2010 to 41.9% in 2017-2018 [33]. Furthermore, over a 20 year period (1988-2018), non-Hispanic Black women showed the greatest increase in obesity [33].

**Figure 1:**
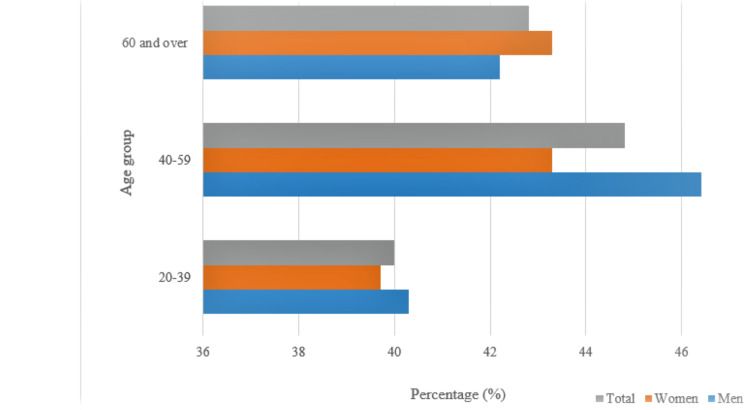
Prevalence of obesity in the United States (2017-2018) among adults aged 20 and over, organized by age and sex Adapted from: NCHS, National Health and Nutrition Examination Survey, 2017–2018 [34]

**Figure 2:**
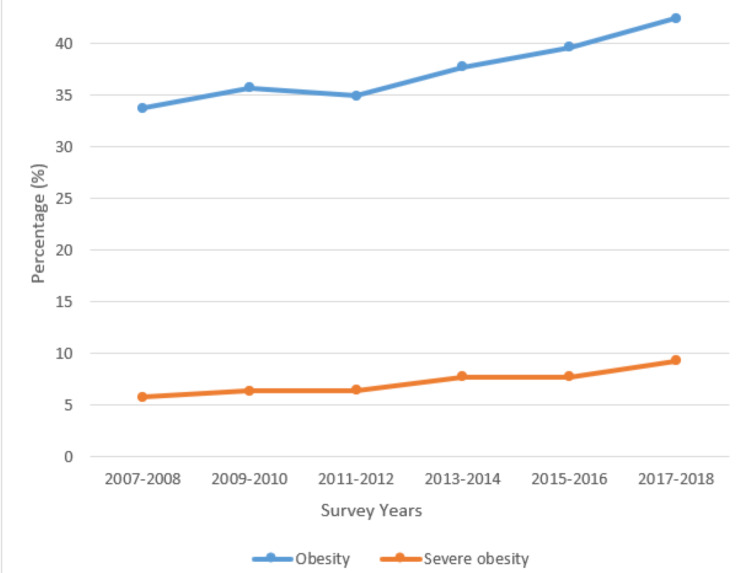
Age-adjusted obesity and severe obesity prevalence trends in the United States over a 10-year period (2007-2008 to 2017-2018) among adults aged 20 and over Adapted from: NCHS, National Health and Nutrition Examination Survey, 2017–2018 [34]

The most recent estimates of the prevalence of obesity were obtained from the National Center of Health Statistics Survey of 2017-2018 which suggest the total prevalence of obesity (BMI>30) is greater than 40% across all age groups (ages 20 and over) in both males and females. The reported total prevalence of adults classified as overweight was 73.6% (BMI 25-29), while 9% were severely obese (BMI>40). Figure 1 depicts the most recent estimates of the prevalence of obesity gathered in 2017-2018 by the National Health and Nutrition Examination Survey. The data are divided into age groups, with the youngest being 20-39 and the eldest being 60 and over. It was found that the prevalence of obesity was higher in men aged 20-39 and 40-59, while women aged 60 and over also had a higher prevalence of obesity [33]. Figure 2 displays data collected from the same survey and illustrates a significant linear trend in both obesity and severe obesity over a 10-year period (P<0.05).

A group of researchers has recently suggested that by 2030, almost one in two adults will have obesity (48.9%) [35]. They also speculate that the prevalence will be greater than 50% in 29 states and no lower than 35% in any state. Their findings also suggest that by 2030, one in four adults is predicted to have severe obesity (24.2%), of which the prevalence will be greater than 25% in approximately 25 states. They also project that severe obesity will likely become the most common BMI category amongst women, low-income adults, and non-Hispanic black adults (27.6%, 31.7%, and 31.7%, respectively) [35]. Another study by Wang et al. had similar findings. They noticed a strong upwards trend in obesity since 1999 in both men and women and found adult obesity prevalence differed across regions and ethnicities from 2011 to 2016, with the South (32%) and Midwest (31.4%) regions of the United States being higher in the prevalence of obesity. In 2016, they found all states had exceeded a prevalence of >20%. The 2016 findings also suggested Black individuals had a >30% prevalence of obesity across 44 states, Hispanics in 32 states, and Whites in 18 states [36]. The growing rise in obesity represents an ongoing concern for the health of the population, with wide disparities across regions and ethnicities.

Inflammation and Its Markers

Inflammation is an integral aspect of homeostasis in the body. Inflammatory factors such as chemokines, cytokines, eicosanoids, and biogenic amines play an important role in different biological processes, ranging from alterations in body temperature to local vascular responses. Inflammatory signal activity can be characterized with regard to its homeostatic effects on the body as opposed to the complexity or diversity of its functions. The markers TNF- and IL1b, in particular, are involved in the inflammatory process, and able to either directly stimulate or inhibit the functions of various homeostatic mechanisms which include activating lipolysis, inhibiting gluconeogenesis, and increasing vascular permeability [37]. Inflammation leads to the increase in the production of pro-coagulative factors, promotes the expression of tissue factor (TF) on white blood cells and endothelial cells, and down-regulates anticoagulant mechanisms [38]. Cytokines, adhesion molecules, and chemokines aid inflammation [39]. Specifically, the chemokine (C-X-C motif) ligand (CXCL) subfamily of cytokines plays a role in inflammatory diseases [40]. A study by Kochumon et al. found CXCL expression was higher in the adipose tissue of obese individuals compared to lean individuals (p<0.05) and correlated positively with BMI (p<0.05) [40]. Moreover, CXCL9, CXCL10, and CXCL11 expression were correlated with pro-inflammatory markers but not anti-inflammatory markers. Furthermore, the expression of CXCL11 was associated with C-reactive protein, causing the subfamily to be a potential biomarker for the onset of adipose tissue inflammation in obesity. Furthermore, inflammation can also influence the process of thrombus dissolution and, as a result, cause fibrosis and thickening of the vein wall [41]. This further supports the notion that inflammation plays a significant role in the homeostatic mechanism, and inflammatory markers may be able to provide a more detailed insight into the pathophysiology behind several diseases. The adipose tissue of obese individuals is infiltrated with pro-inflammatory T-cells and macrophages and causes the accumulation of tumor necrosis factor, α, IL-17, IL-6, and interleukin-1β (IL-1β). On the contrary, in lean individuals, M2 macrophages, Th2, and T-regulatory cells which produce anti-inflammatory cytokines such as IL-10, IL-5, and interferon-γ, are present in the adipose tissue [24].

Obesity and Inflammatory Markers

Obesity, a chronic low-grade systemic inflammation or "metabolic inflammation," is involved in the pathogenesis of many disease processes such as atherosclerosis, coronary artery disease, etc. [4,42,43]. Adipose tissue is a metabolically active endocrine organ responsible for regulating energy expenditure and appetite, along with reproductive and endocrine functions, inflammation, immunity, and serving as a reservoir of triacylglycerol [44]. Visceral adiposity is strongly correlated with a higher risk of diabetes and cardiovascular diseases as compared to a high BMI. Although the reason for this correlation remains unclear, it has been hypothesized that visceral fat is involved in causing systemic inflammation due to its direct access and secretion of free fatty acids and inflammatory cytokines into the portal circulation.

Role of Macrophages, B-cells and T-cells in Obesity

The most abundant immune cells in the adipose tissue of humans are thought to be macrophages. In particular, the M1 phenotype is considered to be a major driver of adipose tissue inflammation [45]. In obese individuals, inflammation of the adipose tissue is linked to a shift in frequency from M2 macrophages (anti-inflammatory) to M1 macrophages (pro-inflammatory), which is also correlated with insulin resistance. Obesity is often linked to the enlargement of adipocytes, which increases the distance between surrounding adipose tissue vasculature and adipose tissue cells, leading to hypoxic conditions [46]. Outcomes associated with this change include adipose tissue fibrosis and infiltration of macrophages to scavenge necrotic adipocytes, which subsequently trigger a local inflammatory response and the production of TNF-a and IL-6 [46]. However, adipose tissue also contains other immune cells such as B-cells, T-cells, dendritic cells, and neutrophils, which also play a role in modulating inflammation. In obese individuals, B-cells have been observed to produce various cytokines, infiltrate adipose tissue, and produce IgG autoreactive antibodies that trigger local inflammation as well as support Th17 function, which promotes a pro-inflammatory T-cell ratio [45-47]. B-cells also play a role in obesity-associated T-cell activation by presenting antigens to T-cells that activate both dendritic cells and macrophages. However, regulatory B-cells and B-1 cells have also been associated with inhibiting inflammation via the production of IL-10 and IgM, respectively. In recent studies, tissue-associated immune cells, in particular T-cells, have been discovered to play a contributing role in obesity-associated inflammation [46]. A 2019 review conducted by Liu et al. further analyzed the role of specific immune cells in obesity [46]. The study found CD8+ T-cells were rarely present in visceral adipose tissue compared to CD4+ T-cells. In comparison to subcutaneous adipose tissue, visceral adipose tissue contains a higher number of CD8+ T cells in obese individuals compared to lean subjects, and more notably, the number of CD8+ cells positively correlated with the subject's BMI [46]. A higher frequency of CD4+ Th17 cells has also been found in visceral adipose tissue. Th17 cells may be increased in number by the body due to adipokines or due to activated production of IL-17 in morbidly obese individuals via circulating memory CD4+ T-cells. T-regulatory cells are predominantly found in lean subjects as they are an anti-inflammatory T-cell type. However, in obese individuals, the number of T-regulatory cells present in adipose tissue was lower. Th2 cells are rarely present in adipose tissue compared to other T-cell subsets. However, they are significantly greater in subcutaneous adipose tissue compared to visceral adipose tissue. Th1 cells are present in greater amounts in visceral adipose tissue, approximately 10-20-fold compared to T-regulatory cells, Th17, or Th2, and are correlated with common plasma inflammatory markers such as IL-6 and CRP.

Role of Hormones in Obesity

The relationship between obesity and chronic low-grade inflammation is not entirely understood, but different mechanisms have been proposed. Several studies suggest adipose tissue can secrete approximately more than 50 hormones and signaling molecules referred to as adipokines [43,48]. Adipokines play an essential role in immunity and the metabolism of glucose [48]. Adipose tissue of lean individuals has been well described as secreting anti-inflammatory adipokines such as IL-4, IL-10, IL-13, IL-1 receptor antagonist (IL-1Ra), transforming growth factor-beta (TGF𝛽), adiponectin, and apelin. On the contrary, adipose tissue of obese individuals predominantly secretes pro-inflammatory cytokines such as IL-6, TNF-𝛼, resistin, visfatin, leptin, angiotensin II, and plasminogen activator inhibitor 1 [43]. The increased production of cytokines in obesity is not yet fully understood. However, it is speculated that there are mechanisms within the enlarged, lipid-loaded adipocytes responsible for maintaining and restoring energy homeostasis in cases of excessive nutrient intake. These regulatory mechanisms act to control the local production of cytokines and arrest the storage of lipids within the hypertrophied adipocyte. This can become problematic in states of sustained obesity due to a chronic systemic inflammatory response.

Leptin is a hormone that acts to regulate appetite and energy balance [49]. Leptin’s role in metabolism is of importance as it is one of the first proteins secreted by adipose tissue [38], and primarily secreted in proportion to fat mass [50]. Leptin exerts several effects on energy balance, such as increasing energy expenditure through its effects on the hypothalamus, influencing the metabolism of lipids and glucose, altering neuroendocrine function, and decreasing food intake. Obese individuals present with increased leptin levels with little to no impact on energy homeostasis. This gives rise to a paradox regarding leptin resistance in obese individuals that is not yet well understood. Studies involving both obese humans and rodents showed that leptin resistance may have a direct contribution to the reduction of lipid oxidation in organs sensitive to insulin, which may lead to lipid accumulation and insulin resistance [51,52]. The influence of leptin on insulin resistance is also not yet fully understood. In low-insulin states such as diabetes, leptin is decreased and increases after insulin treatment [53]. In humans, insulin resistance is associated with elevated serum leptin levels, independent of body mass [54]. Leptin levels are significantly low and correlate with insulin resistance markers in individuals with lipodystrophy [55], a condition defined by a lack of adipose tissue [56]. Leptin therapy in these patients is associated with an improved metabolic state and significant improvements in insulin sensitivity, which suggests that leptin can act as a signal in the body and promotes the regulation of the body's sensitivity to insulin [57].

Adiponectin is a hormone produced by adipocytes. In obesity, serum levels of adiponectin are found in low amounts, unlike leptin [58]. Adiponectin has been shown to have insulin-sensitizing effects. In adiponectin-deficient transgenic mice, improved insulin sensitivity was observed [59], and several other studies have consistently associated insulin sensitivity with plasma adiponectin levels in rodent and human models [60].

Leptin, adiponectin, resistin, chemerin, visfatin, IL-1, IL-6, IL-8, IL-10, TNF-a, plasminogen activator inhibitor 1, retinol-binding protein-4, and monocyte chemoattractant protein-1 are all adipokines involved in the resistance to insulin.

Established and novel obesity-related biomarkers include those of glucose-insulin homeostases such as insulin, proinsulin, C-peptide, and insulin-like growth factors; biomarkers of adipose tissue such as leptin, resistin, apelin, adiponectin, omentin, and fatty-acid-binding protein-4; inflammatory biomarkers such as CRP, TNF-α, IL-6, and omics-based biomarkers such as microRNAs and metabolites [61].

Fatty Acids Can Induce Inflammation

Obesity commonly presents with increased levels of lipids in the blood, including non-esterified fatty acids (NEFA) [62]. The inflammatory response can be triggered by the chemical nature of these fatty acids. Adipose tissue has a limited capacity to store energy, and once this is exceeded, lipolysis occurs within the adipocytes, causing a release of NEFA into the circulation. Once in circulation, NEFA can accumulate and exert toxic effects on other tissues and organs, causing insulin resistance and cell death, a phenomenon referred to as lipotoxicity. Previous studies on weight-discordant twins showed the adipose tissue of obese twins displayed signs of insulin resistance and an active inflammatory and immune response [63]. It has also been suggested that fatty acids can regulate adipokine production and secretion [64,65]. Alternatively, it is proposed that through the activation of cell receptors such as peroxisome proliferator-activated receptors (PPAR), NEFA may act as natural ligands and directly induce the inflammatory pathway, causing upregulation of the NF-kB transcription factors, essential in the initiation of the inflammatory response. In addition, it is hypothesized that PPAR may be involved in the phenotypic switch of adipose tissue macrophages from M2 (anti-inflammatory) to M1 (pro-inflammatory) [66].

Amino Acids and Obesity

The relationship between amino acids and obesity is one that has not yet been fully explored. A study by Mikkola et al. sought to understand the association between circulatory amino acids and fat and lean body mass [67]. A higher fat mass was associated with greater amounts of all branched-chain amino acids, aromatic amino acids, and alanine in both men and women (p<0.008). Similarly, a higher lean mass was associated with greater amounts of all branched-chain amino acids in both men and women (p<0.001). However, in men, lean mass was inversely associated with the level of glycine (p<0.001). Other research has found a positive association between blood levels of branched-chain amino acids and obesity, which may be due to their relationship with insulin resistance [68]. Overall, circulating amino acids may be useful as a predictor of obesity in older adults [68].

Resistin and Its Association With Obesity

Steppan and Lazar studied the role of resistin as an antagonist to insulin in both in vivo and in vitro conditions [69]. Their findings indicated resistin was present in greater amounts in diabetic and obese mice. The exogenous administration of resistin has been observed to increase plasma glucose levels as well as its endogenous production in rodents [70,71]. Mononuclear cells in humans, such as macrophages, and both adipocytes and macrophages in rodents, produce resistin [71,72]. Other chemokines such as IL-6, TNF- α, visfatin, MCP-1, and PAI-1 are expressed not only in adipocytes but also in activated macrophages and other immune cells. The relative amounts produced by the adipocytes compared to the macrophages in adipose tissue are still unknown. More recently, the potential role of resistin as a mediator between adipose tissue, inflammation, immunity, and adipose tissue has been reviewed [72]. There is also evidence that suggests resistin acts as a pro-inflammatory adipocytokine which is enhanced during strenuous activity such as running marathons and is associated with cartilage degradation markers [73].

Several studies have suggested resistin plays a role in inflammatory conditions due to its secretion by mononuclear cells [74-78]. In patients with obstructive sleep apnea, resistin levels are thought to be correlated with cell adhesion molecules (ICAM1) and positively associated with other inflammation markers such as lipoprotein-associated phospholipase and soluble TNF-R type II in patients with atherosclerotic plaques [74,75]. Lipopolysaccharide has also been found to induce the expression of resistin in human and mouse macrophages. Through the NFκB pathway, a cascade involving pro-inflammatory cytokine secretion, resistin is induced by TNF and can further induce the production of IL-6 in peripheral blood mononuclear cells in humans [76,77]. Moreover, changes in resistin are significantly correlated with visfatin and IL-6 levels [78].

Visfatin and Its Relationship With Obesity

Visfatin, a novel cytokine originating from adipose tissue, was identified by Fukuhara et al. [79]. Due to its high expression in visceral fat cells, once secreted, visfatin acts as a protein mediator, similar to the enzyme (Nicotinamide phosphoribosyl transferase) NAMPT, involved in the NAD+ salvage pathway. At first, visfatin was identified as a pre-B cell colony enhancing factor (PBEF), secreted by peripheral blood lymphocytes in humans [80]. The insulin-mimetic effect of visfatin is predominant in the liver, bone marrow, and skeletal muscle, where it acts as a growth factor for B-lymphocyte precursors [79,81]. As mRNA levels of visfatin increase during the differentiation process of adipocytes, visfatin levels in the body are closely correlated with WAT accumulation. Visfatin synthesis is regulated by many factors, including IL-6, TNF, glucocorticoids, and growth hormone. Visfatin is predominantly produced by WAT and endotoxin-challenged neutrophils. These neutrophils then prevent the apoptosis of cells through a caspase 3 and 8 mediated process [81]. Patients with inflammatory bowel diseases have elevated levels of circulatory visfatin and increased levels of visfatin mRNA present in their intestinal epithelium. [82] It is also noted that visfatin can induce the chemotaxis and production of TNF, IL-1b, IL-6, and other costimulatory molecules via CD14+ monocytes, as well as increase their ability to generate alloproliferative responses mediated intracellularly by MEKI and p38 in lymphocytes [82]. Greater circulating levels of visfatin have been seen in patients with rheumatoid arthritis [83] and acute lung injury [84]. Significantly higher visfatin mRNA expression was also found in inflammatory bowel disease [82]. The binding affinity of visfatin/PBEF/Nampt to the insulin receptor was similar to that of insulin [79]. Previous research has demonstrated higher levels of visfatin in diseases such as diabetes mellitus [85-87]. However, when an experiment was performed on a cohort of obese patients, there was no correlation observed between PBEF/visfatin and glucose infusion [88]. The binding of visfatin to insulin receptors and its insulin-mimetic activity remains controversial. However, recent research has found systemic NAD+ biosynthesis mediated by Nampt/visfatin is essential for proper β cell function [89]. These findings further support the role of visfatin in the regulation of glucose homeostasis [89]. Children with a higher BMI have increased levels of visfatin, indicating the implication of this new adipokine in the inflammatory mechanisms of obesity beginning in early childhood [90]. Higher levels of visfatin were also found to be higher in females with visceral obesity and morbidly obese subjects after gastric banding [79]. In contrast, morbidly obese women who had lost more than 20% of their BMI showed a decrease in circulating visfatin. Overall, these studies depict that the greater the BMI (obesity), the higher the concentration of visfatin is present, and visfatin levels tend to decrease following weight loss.

Interaction Among Inflammatory Markers in Obesity

The prevalence of obesity has increased significantly over the past decade, contributing to the state of the epidemic in the United States. The etiology of obesity is multifactorial, with some causes including genetic, environmental, behavioral, socioeconomic, and psychological contributions. Obesity is regulated by complex interactions between the central nervous system and endocrine tissues, which result in a chronic positive energy balance [91,92]. Adipocytes produce and secrete several proteins, collectively called adipokines, which play important roles in inflammation. TNF-α, leptin, IL-6, adiponectin, resistin, and visfatin are examples of adipokines.

Vendrell et al. conducted a study on 57 morbidly obese white subjects (mean age of 42.2±9.2 years; 49 women) and 117 non-morbidly obese subjects (mean age 49.2±12.4 years; 89 women) [93]. Analysis of these subjects showed that those classified as morbidly obese had generally higher values across all anthropometric measurements (BMI, waist-to-hip ratio, and body fat) as compared to the non-morbidly obese subjects. The results indicated there were significant differences observed in leptin levels (13.3±7.0 vs. 34.2±13.4 ng/mL, p<0.001) and adiponectin levels (11.3±4.5 vs. 18.0±6.7 g/mL, p<0.001) between men and women in the non-morbidly obese group. There was also a significant gender difference for resistin. The researchers also found a positive correlation between leptin and BMI in nonmorbidly obese subjects (r=0.390, p<0.01) while adiponectin displayed a strong negative correlation with weight (r=0.33, p=0.001), fasting insulin (r=0.28, p=0.05), and triglycerides (r=0.22, p=0.006); however, a positive correlation with HDLc (r=0.36, p=0.001) was observed. A positive association between serum resistin and sTNFR1 (r=0.31, p=0.01) and triglycerides (r=0.24, p=0.01) was also reported. In the morbidly obese group, adiponectin, leptin, and ghrelin were not correlated with any metrics of body composition. A significant and positive correlation was found between resistin and weight (r=0.48, p<0.001), fat-free mass (r=0.39, p 0.01), and BMI (r=0.39, p<0.005). Findings from the bivariate correlation were further analyzed using multivariate analysis to allow for the control of potential confounders. Upon adjustment for age, gender, and BMI in the nonmorbidly obese patient group, there was a positive association between leptin and gender (β=0.52, p<0.001) and BMI (β=0.24, p=0.009). Furthermore, adiponectin was positively correlated with gender (β=0.22, p=0.05) and HDLc (β=0.31, p=0.007), and negatively correlated with weight (β= −0.38, p<0.001). After adjusting for these variables, circulating resistin levels were positively associated with sTNFR1 (β=0.28, p=0.007), leading to the loss of correlation with triglycerides previously observed in the bivariate analysis. No adipokines were found to be associated with metabolic or clinical variables in the morbidly obese subjects, with the exception of resistin and ghrelin levels that exhibited a positive correlation with sTNFR2 upon adjusting for age, gender (β=0.41, p=0.008), and BMI (β=0.33, p=0.04).

## Conclusions

Based on this review, we can conclude that multiple organ systems, especially those of adipose tissue and muscle, are responsible for metabolic homeostasis. Adipocytes secrete adipokines, which are hormones that regulate metabolism by acting on multiple cells and organs. The amounts of these hormones present in children, adolescents, and adults (including elderly and overweight/obese people), along with the role they play in obesity, diabetes, hypertension, metabolic syndrome, atherosclerotic diseases, etc., are of significance. Thus, in order to gain a deeper insight into the regulation and pathology of these adipokines/hormones, it is important to further explore how these factors affect lifestyles such as regular exercise, dietary intervention, supplementation, or how a combination of these may influence their concentrations in the body. Although the roles of resistin and visfatin in homeostasis/metabolism are not yet completely understood; the interest in further researching these hormones should remain high. Several research studies have shown there is an association present between abdominal and total obesity and various inflammatory markers. However, this association has not been fully comprehended. Obesity is a chronic low-grade inflammation associated with an increase in inflammatory markers. Future research will provide insights into the correlation of different inflammatory markers with one another in different populations, to aid in the further understanding of the underlying mechanism between obesity and inflammation.
